# VALIDITY AND RELIABILITY OF REMOTE, SMARTPHONE-BASED ASSESSMENT OF DUAL-TASK STANDING AND WALKING IN OLDER ADULTS

**DOI:** 10.1093/geroni/igz038.034

**Published:** 2019-11-08

**Authors:** Brad Manor, Wanting Yu, On-Ye Lo, Hao Zhu, Thomas G Travison, Lewis Lipsitz, Junhong Zhou

**Affiliations:** 1 Hinda and Arthur Marcus Institute for Aging Research, Harvard Medical School, Boston, Massachusetts, United States; 2 Hinda and Arthur Marcus Institute for Aging Research, Roslindale, Massachusetts, United States

## Abstract

Dual task walking assessments provide valuable insights into cognitive-motor function in aging. To date, such assessments have been limited primarily to laboratory-based settings. We thus created a smartphone App utilizing multi-media instructions and the phone’s motion sensors to record movements during normal and dual task walking, with the phone placed in the user’s pants pocket. Thirty younger and older adults completed two lab visits, during which walking data were simultaneously acquired by the App and the GAITRite mat. Participants also completed App-based assessments in their homes on three separate days. Across all detected strides in laboratory trials, gait metrics derived from the App correlated closely with those derived from the GAITRite mat (r2>0.96). Across trials, gait metrics demonstrated excellent test-retest reliability, both within and between laboratory visits and home-based assessments (ICC: 0.79–0.90). Remote, smartphone-based dual task walking assessments may therefore be feasible for relatively healthy younger and older adults.

